# Linear-dendritic block copolymers as a green scale inhibitor for calcium carbonate in cooling water systems

**DOI:** 10.1080/15685551.2017.1296530

**Published:** 2017-02-23

**Authors:** Guangqing Liu, Mengwei Xue, Qinpu Liu, Yuming Zhou

**Affiliations:** ^a^ School of Environmental Science, Nanjing Xiaozhuang University, Nanjing, P.R. China; ^b^ School of Chemistry and Chemical Engineering, Southeast University, Nanjing, P.R. China

**Keywords:** Linear-dendritic block copolymers, green, scale inhibitor, calcium carbonate, cooling water systems

## Abstract

Water-soluble monomer APEG-PG-(OH)n were produced and the Structure of APEG-PG-(OH)5 were identified by ^1^H-NMR. APEG-PG-(OH)n were copolymerized with maleic anhydride (MA) to synthesize no phosphate and nitrogen free calcium carbonate inhibitor MA/APEG-PG-(OH)n. The structure and thermal property of MA/APEG-PG-(OH)5 were characterized and measured by ^1^H-NMR, GPC and TGA. The observation shows that the dosage and *n* value of MA/APEG-PG-(OH)n plays an important role on CaCO_3_ inhibition. MA/APEG-PG-(OH)5 displays superior ability to inhibit the precipitation of calcium carbonate, with approximately 97% inhibition at a level of 8 mg/L. The effect on formation of CaCO_3_ was investigated with combination of SEM and XRD analysis.

## 1. Introduction

For environmental and economic reasons, a greater number of cycles for industrial water should be used. However, it cannot be realized without development of scale control methods [1–6]. The potential of mineral precipitation continues to be by far the most costly design and an operating problem in recycling water systems [7,8]. Commonly, mineral precipitation consist of calcium scales, zinc scales, magnesium hydroxide, ferric hydroxide, barium sulfate, etc., among which calcium carbonate scales are considered most frequent in cooling water systems [9–13].

The most common and effective method of scale controlling is the use of chemical additives as scale inhibitors that retard or prevent scale formation even in very small concentrations [7,12]. At present, Amino Trimethylene Phosphonic Acid (ATMP) and 2-Phosphonobutane-1,2,4-Tricarboxylic Acid (PBTCA) are well known scale inhibitors in cooling water systems. Although the nitrogen, and phosphorus containing scale inhibitors are highly efficient, their use is limited because these compounds are nutrients for algae, which has the potential to ruin the environment [14]. As a result, the current trend for inhibitors use is towards more environmental-friendly ‘green’ chemicals that are undoubtedly the trend of development [15–17].

In our previous work, no phosphate and nitrogen free scale inhibitor (AA-APEL) which has a superior calcium tolerance were prepared from allyloxy polyethoxy ether and Acrylic acid [12]. But Acrylic acid is more expensive than maleic anhydride.

On the basis of all these information, polyether-typed scale inhibitor, linear-dendritic double hydrophilic block copolymer (MA/APEG-PG-(OH)n) was synthesized. In comparison with traditional scale inhibitor, MA/APEG-PG-(OH)n derived from capped polyether, easily prepared with non-toxic, biodegradable, lower cost, reliable reproducibility and less dosages, have superior scale inhibitive performances. In addition, MA/APEG-PG-(OH)n belongs to an environment friendly scale inhibitor, containing elements of carbon (C), hydrogen (H), oxygen (O) and is non-phosphorous (P) and nitrogen (N) free.

## 2. Experimental

### 2.1. Materials

Allyloxy poly(ethylene glycol) (APEG) was supplied by Jianghai Environmental Protection Co., Ltd. (Changzhou, Jiangsu, P.R. China). Glycidol (99%) was purchased from Aladdin Chemistry Co., Ltd. (Shanghai, P.R. China). Other reagents such as maleic anhydride, potassium peroxydisulfate, ammonium persulfate, of AR grade were obtained from Zhongdong Chemical Reagent Co., Ltd. (Nanjing, Jiangsu, P.R.China). Commercial inhibitors were technical grade and supplied by Jianghai Environmental Protection Co., Ltd. (Changzhou, Jiangsu, P.R. China). Distilled water was used in all the studies.

### 2.2. Measurements

^1^H NMR spectra were recorded on a Mercury VX-500 spectrometer (Bruker AMX500) using tetramethylsilane (TMS) internal reference and deuterated dimethyl sulfoxide (DMSO-d6) as a solvent. Thermogravimetric analysis (TGA) was performed on samples at temperatures ranging from 25 to 600 °C. Such signals were obtained at a heating rate of 20 °C/min in air using a Perkin-Elmer Derivatograph instrument. Molecular weight of the polymers was investigated through gel permeation chromatography (GPC-Waters-2410).

### 2.3. Synthesis of APEG-PG-(OH)n (*n* = 3, 5, 7, 9, 11)

**Scheme 1.**
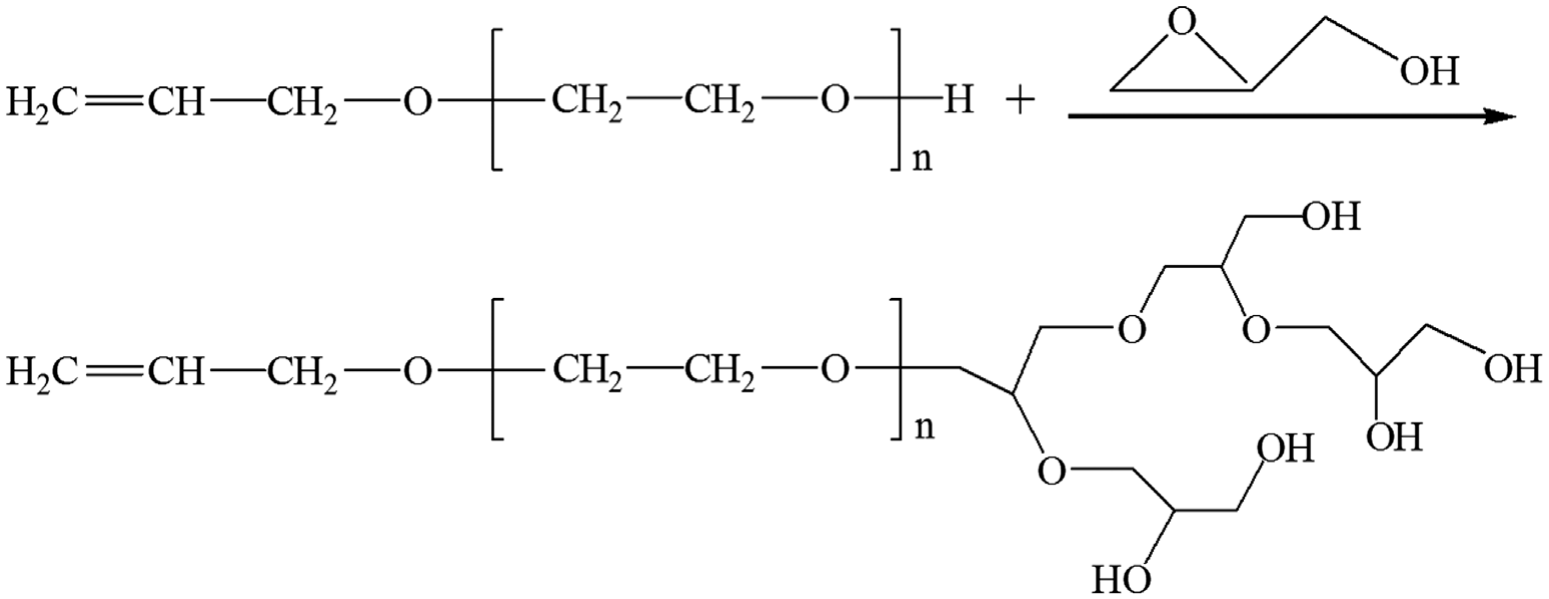
Synthesis of APEG-PG-(OH)5.

Synthesis of APEG-PG-(OH)n (n is the number of hydroxyl) were carried out in a reactor equipped with a mechanical stirrer and dosing pump under nitrogen atmosphere. 24 g of APEG was partially deprotonated (30%) with potassium methylate solution by distilling off excess methanol from the melt. A certain quantity of glycidol was slowly added at 75 °C, choosing the initiator amount according to the monomer/initiator ratio [18]. The reaction mixture was heated to 85 °C and maintained at this temperature for a further 2.5 h, to ultimately obtain yellowish viscous liquid APEG-PG-(OH)n. The synthesis procedure of APEG-PG-(OH)5 is shown in Scheme 1.

### 2.4. Synthesis of MA/APEG-PG-(OH)n (*n* = 3, 5, 7, 9, 11)

**Scheme 2.**
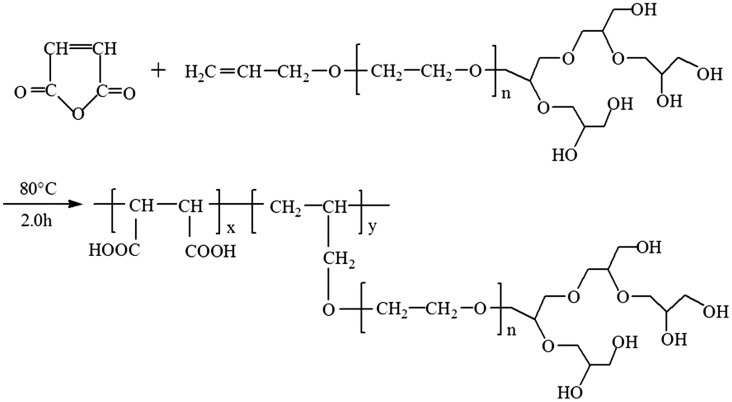
Synthesis of MA/APEG-PG-(OH)5.

A 4-neck round-bottom flask equipped with a thermometer and a magnetic stirrer was charged with 20 mL of distilled water and 10 g of MA, and heated to 65 °C with stirring under nitrogen atmosphere. Subsequently, 10 g of APEG-PG-(OH)n in 20 mL of distilled water (APEG-PG-(OH)n:MA mass ratio = 1:1) and the initiator solution (0.2 g of ammonium persulfate in 20 mL of distilled water) were added separately at constant flow rates over a period of 1.5 h. The reaction mixture was heated to 80 °C and maintained at this temperature for a further 2.0 h, ultimately afford an aqueous copolymer solution containing approximately 35% solid. The synthesis procedure of MA/APEG-PG-(OH)5 from MA and APEG-PG-(OH)5 is shown in Scheme 2.

### 2.5. Precipitation of calcium carbonate experiments

All precipitation experiments were carried out in flask tests and all inhibitors dosages given below are on a dry-inhibitor basis. Tests of the inhibitors were carried out using supersaturated solutions of CaCO_3_ at 60 °C. The solutions were prepared by dissolving in distilled water reagent grade CaCl_2_ and NaHCO_3_ (Zhongdong Chemical Reagent Co.) at equivalent concentrations of 24 milli-equivalent/L (cooling water code GB/T 16632-2008). The supersaturation level of the solutions corresponded to a Langelier Index of 2.1. Each inhibition test was carried out in a flask of 500 mL immersed in a temperature-controlled bath for 10 h. Precipitation of CaCO_3_ was monitored by analyzing aliquots of the filtered (0.22 μm) solution for Ca^2+^ ions using EDTA complexometry as specified in code GB/T 15452-2009. Inhibitor efficiency was calculated from the following equation:

$$\text{inhibition}\left(\%\right)=\frac{{\left[{\text{Ca}}^{2+}\right]}_{\text{final}}-{\left[{\text{Ca}}^{2+}\right]}_{\text{blank}}}{{\left[{\text{Ca}}^{2+}\right]}_{\text{initial}}-{\left[{\text{Ca}}^{2+}\right]}_{\text{blank}}}\times 100\%$$

where [Ca^2+^]_final_ is concentration of Ca^2+^ ions in the filtrate in the presence of inhibitor after calcium carbonate supersaturated solutions were heated for 10.0 h at 60 °C, [Ca^2+^]_blank_ is concentration of Ca^2+^ ions in the filtrate in the absence of inhibitor after calcium carbonate supersaturated solutions were heated for 10.0 h at 60 °C, and [Ca^2+^]_initial_ is concentration of Ca^2+^ ions at the beginning of the experiment.

## 3. Results and discussion

### 3.1. ^1^H NMR measurements

**Figure 1.**
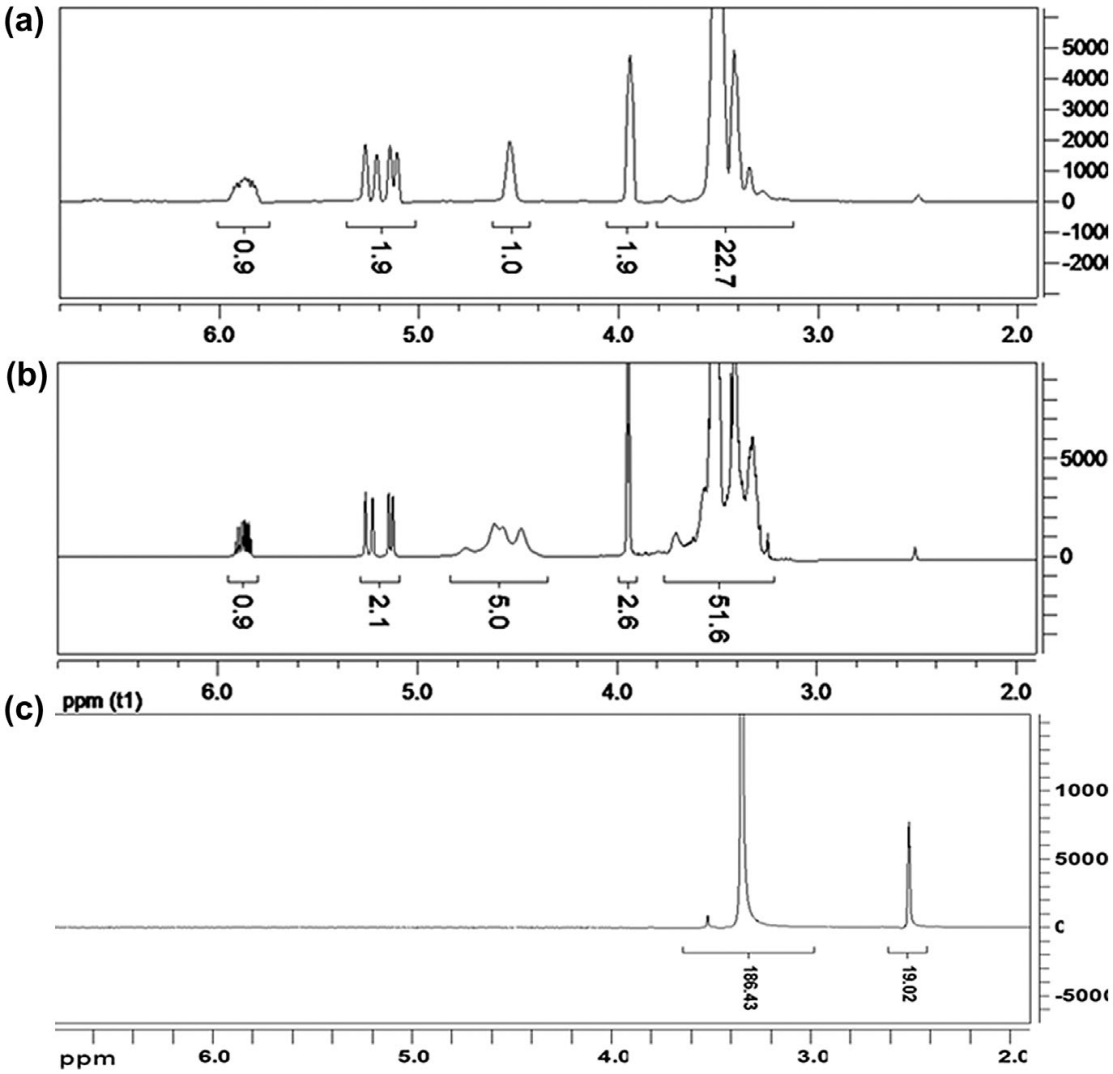
^1^H NMR spectra of APEG (a), APEG-PG-(OH)5 (b) and MA/APEG-PG-(OH)5 (c).

The ^1^H NMR spectra of APEG，APEG-PG-(OH)5 and MA/APEG-PG-(OH)5 are shown in Figure 1. ^1^H NMR spectral analysis reveals that the ^1^H NMR spectra of APEG and APEG-PG are almost the same, except the single peak at 4.5 ppm for active hydroxyl group of APEG (Figure 1(a)); while signals at 4.3–4.8 ppm belong to hydroxyl groups of APEG-PG-(OH)5 (Figure 1(b)). The number of hydroxyl groups was 5, which means that on average 4 glycidol units were grafted to APEG chain. 3.80–6.00 ppm in (b) double bond absorption peaks completely disappeared in Figure 1(c). This reveals that free radical polymerization among MA and APEG-PG-(OH)5 has happened.

### 3.2. GPC analysis

**Figure 2.**
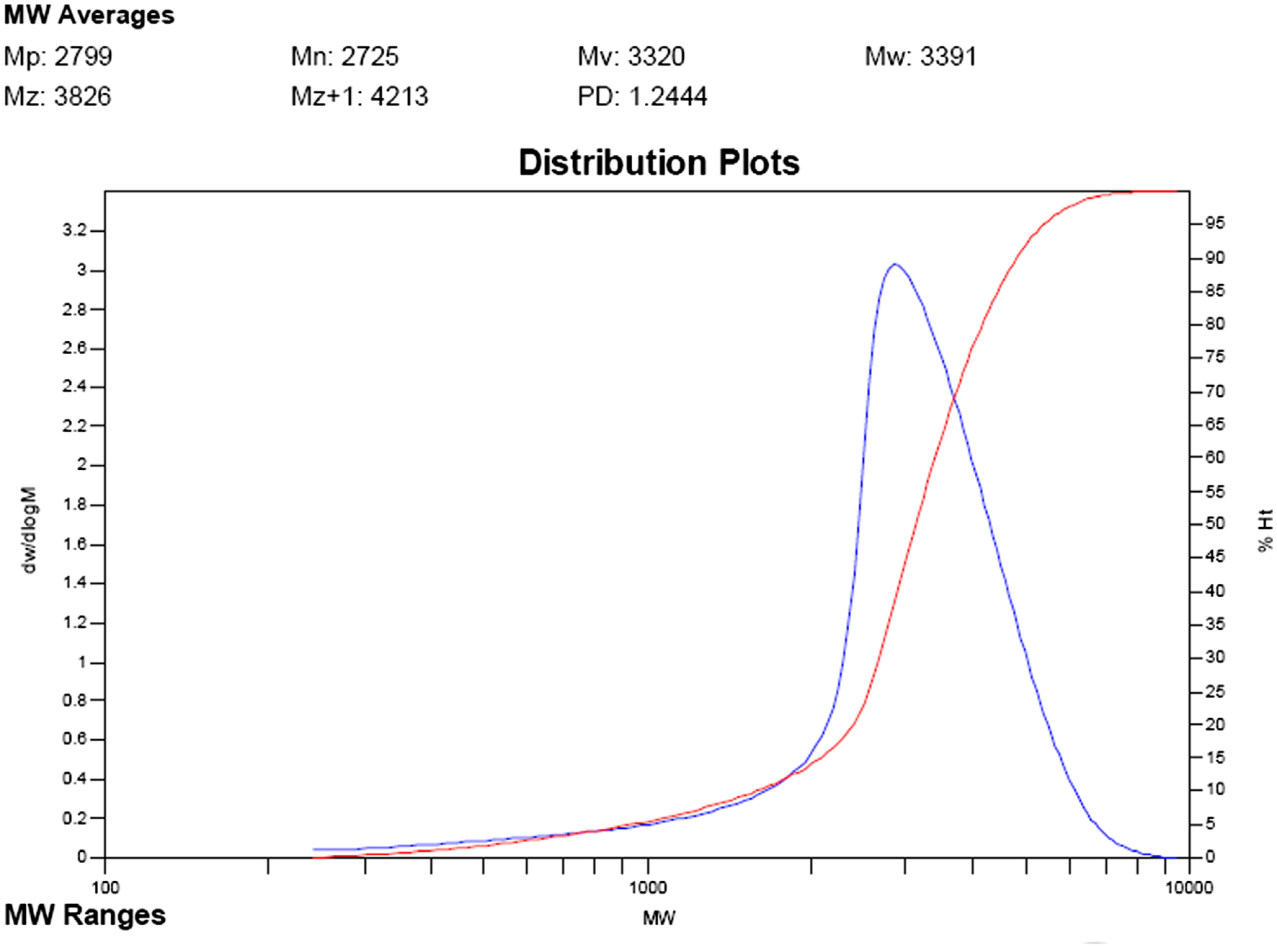
Retention curve profiles of MA/APEG-PG-(OH)5.

The molecular mass distributions of MA/APEG-PG-(OH)5 copolymer was investigated via GPC. The weight-average molecular weight (Mw) is 3391, and the polydispersity index (PDI) is 1.224, which strongly suggests that the monomers satisfactorily undergo copolymerization to produce uniform copolymers. The GPC response curve of MA/APEG-PG-(OH)5 showed in Figure 2 also indicates a typical low molecular weight product of copolymerization. Molar mass at the maximum peak (Mp), viscosity-average molecular weight (Mv) and the z-average molecular weight (Mz) were also obtained in the curve profiles. Their molecular weights are less than 1.0 × 10^4^. Low molecular weight is an essential parameter for efficient scale inhibition which is achieved through careful control of reaction rate and timely termination of chain propagation.

### 3.3. Thermal property of monomers and copolymer

**Figure 3.**
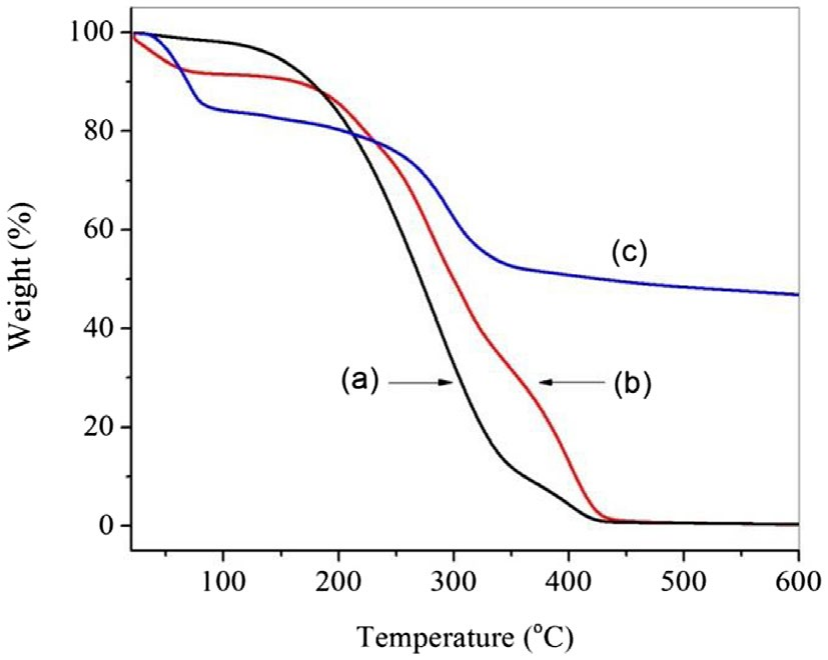
TGA thermograms of APEG (a), APEG-PG-(OH)5 (b) and MA/APEG-PG-(OH)5 (c).

Thermogravimetric analysis (TGA) was used to obtain further information on the structures of APEG, APEG-PG-(OH)5 and MA/APEG-PG-(OH)5 . The corresponding TGA are depicted in Figure 3. The figure shows that degradation of APEG, APEG-PG-(OH)5 and MA/APEG-PG-(OH)5 all proceeded in three or four stages. The first decomposition stage was assigned in the removal of the volatile matter present in these samples, such as entrapped moisture or extraction solvent. The greatest percentage decomposition of APEG and APEG-PG-(OH)5 occurred in the second stage (145–435 °C), as indicated by the corresponding weight loss values(Figure 3(a) and (b)). It may be attributed to cracking and gasification at high temperatures. It is indicated that free radical polymerization can effectively enhance thermal stability of copolymer (Figure 3(c)).

### 3.4. Influence of MA/APEG-PG-(OH)n dosage and *n* value of alcium carbonate inhibition

**Figure 4.**
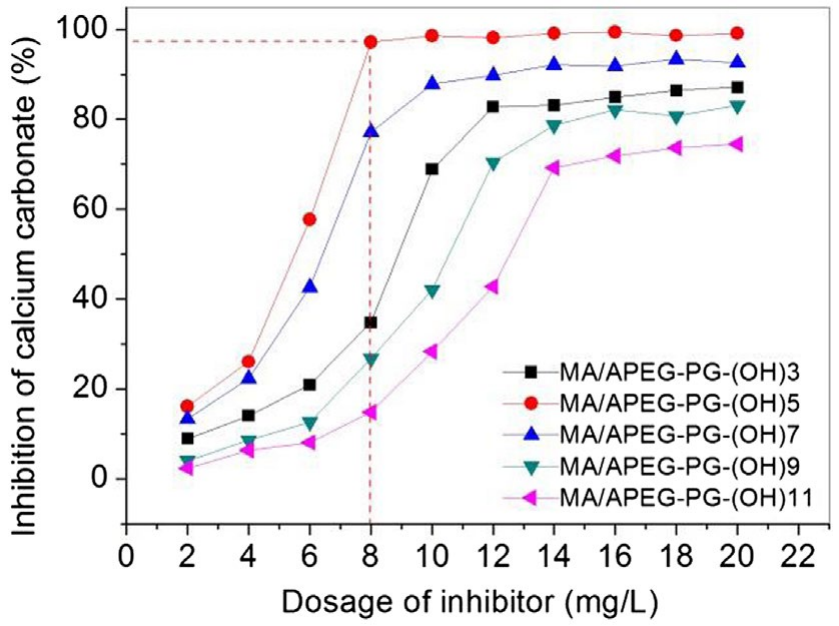
Inhibition rate on calcium carbonate precipitation in the presence of varying dosages of MA/APEG-PG-(OH)n (*n* = 357,911).

The ability of to control calcium carbonate deposits was shown in Figure 4. We can found that MA/APEG-PG-(OH)n have the similar tendency of the dosage on the performance behavior, For example at the dosage of 2–4 mg/L, the polymers show poor calcium carbonate inhibition; in a certain range, scale inhibition effect increases with increasing the copolymer concentration; and when dosage exceeds the threshold, the effect is no longer increase. It has been reported in earlier studies on polymeric threshold inhibitors. It should be noted that the number of the hydroxyl groups also has a great influence on the scale inhibition effect. Compared to the copolymer of MA/APEG-PG-(OH)n (*n* = 37,911), MA/APEG-PG-(OH)5 displays superior ability to inhibit the precipitation of calcium carbonate, with approximately 97% inhibition at a level of 8 mg/L. Threshold dosage of MA/APEG-PG-(OH)5 is much lower than MA/APEG-PG-(OH)n/PA (*n* = 37,911). When the number of OH groups is 5, the weight-average molecular weight (Mw) is 3391, and MA/PEG-PG-(OH)5 displays superior ability to inhibit the precipitation of calcium carbonate. When the number of OH groups gets smaller, branching degree of MA/PEG-PG-(OH)n decline quickly, the MA/PEG-PG-(OH)n adsorption capacity of Ca^2+^ will be relatively reduced, so the inhibition capacity of calcium carbonate decreases. When the number of OH groups gets larger, soluble of MA/PEG-PG-(OH)n decrease greatly, the calcium carbonate inhibition of MA/APEG-PG-(OH)n also decreases dramatically.

### 3.5. Influence of solution property on CaCO_3_ inhibition

**Figure 5.**
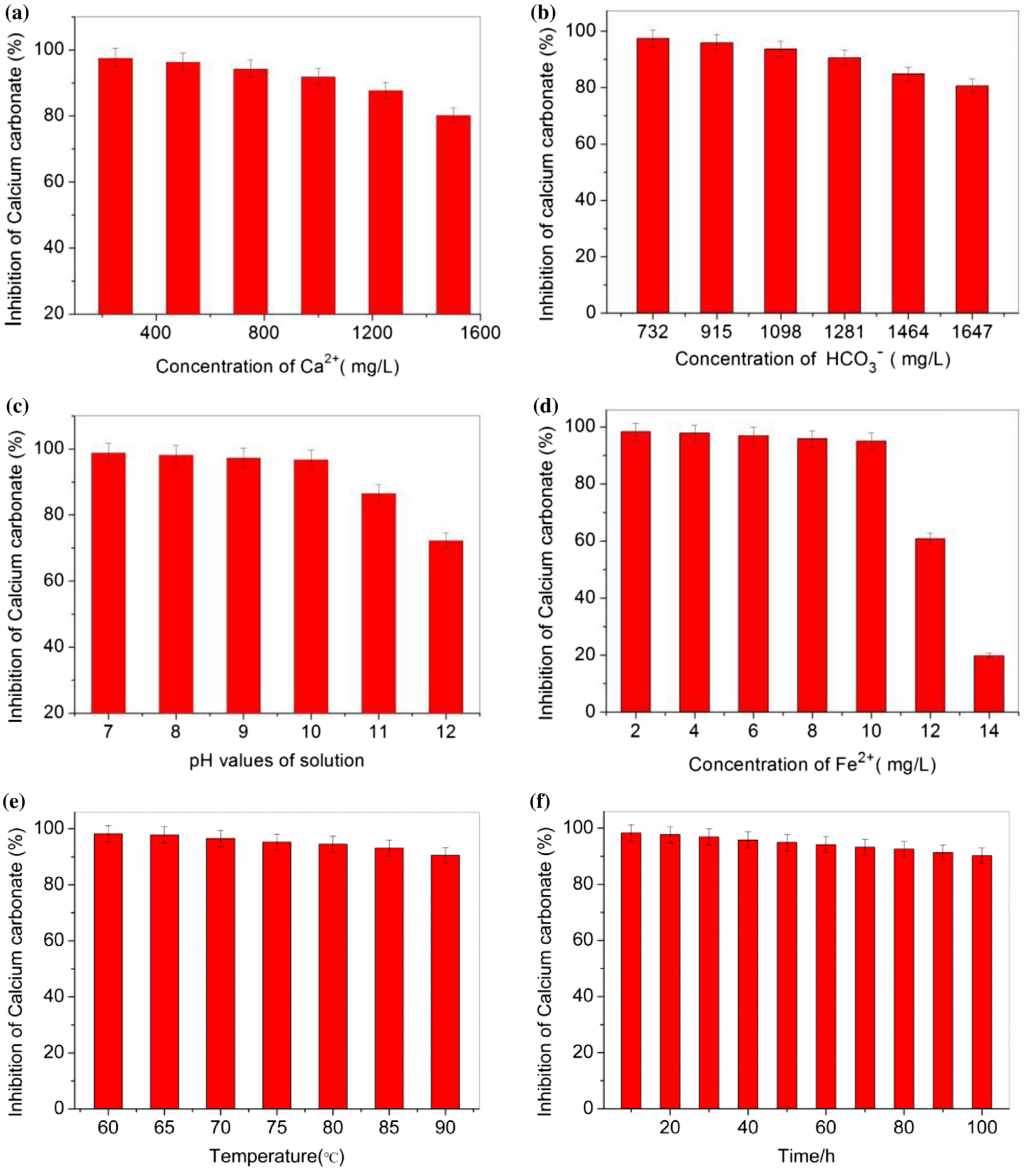
Inhibition at a level of 8 mg/L MA/APEG-PG-(OH)5 as a function of solution Ca^2+^ concentration (a), HCO_3_^−^ concentration (b), pH (c), Fe^2+^ concentration (d), Temperature (e) and Time (f).

Solution properties have a great influence on the precipitation of calcium carbonate. In order to optimize parameters of the recycling water process on an industrial scale, we investigated the effect of the solution parameters on the calcium carbonate inhibition of MA/APEG-PG-(OH)5. The results are shown in Figure 5 as follows:

Figure 5(a) indicates MA/APEG-PG-(OH)5 provides unexceptionable calcium carbonate inhibition under conditions of water with a much higher hardness (HCO_3_^−^concentration kept constant and at 732 mg/L level). Figure 5(b) clearly demonstrates that with HCO_3_^−^concentration increased (Ca^2+^ concentration kept constant and at 240 mg/L level), MA/APEG-PG-(OH)5 polymers also possess excellent calcium carbonate inhibition. As illustrated in Figure 5(c), calcium carbonate inhibitory power drops 24.3% with increasing the solution pH from 7 to 12. The reason is probably that the solubility of calcium carbonate decreases when increasing the pH. At a pH of 8–10, the usual pH values of the industry recycling water, MA/APEG-PG-(OH)5 still shows superior calcium carbonate inhibition.

In consideration of the favorable reaction with iron ions, some antiscalants, such as PMA, would lose most of their effectiveness against calcium carbonate scale in the presence of traceamounts of iron in solutions [19]. The results in Figure 5(d) show MA/APEG-PG-(OH)5 still has excellent calcium carbonate inhibition at levels of 2–10 mg/L iron ions in supersaturated solutions of calcium carbonate. However, the calcium carbonate inhibition of MA/APEG-PG-(OH)5 decreases dramatically at levels of 12–14 mg/L iron ions, and MA/APEG-PG-(OH)5 is totally ineffective against the calcium carbonate scale when the concentrations of iron ions in solutions are 14 mg/L. The trace amounts of iron are usually on the order of 1–5 mg/L in industrial recycling water systems, hence, the copolymer of MA/APEG-PG-(OH)5 is still a excellent antiscalant for calcium carbonate inhibition, even in the presence of trace amounts iron ions in aqueous solutions.

Figure 5(e) clearly demonstrates that the MA/APEG-PG-(OH)5 polymer have good thermal stability. When increasing the solution temperature from 60 to 90 °C, MA/APEG-PG-(OH)5 retains most of its activity, only 7.7% loses in calcium carbonate inhibition. As illustrated in Figure 5(f), calcium carbonate inhibitory power drops only 8.1% with increasing the time from 10 to 100 h. Thus, the incorporation of the high performance scale inhibitor MA/APEG-PG-(OH)5 into recycling water ensures a better overall system performance.

Water cooling systems contains several ions, in order to take into account the interaction of these ions. we investigated the effect of Ca^2+^, Mg^2+^, CO_3_^2−^, SO_4_^2−^ and PO_4_^3−^ ions on the calcium scales inhibition of MA/APEG-PG-(OH)5. The results are shown in Table 1 as follows: The data in Table 1 show that, under the experimental conditions of 240 mg/L Ca^2+^, 732 mg/L CO_3_^2−^, pH 9.0, 80 °C, and 8 mg/L antiscalants, when the concentrations of Mg^2+^, PO_4_^3−^ and SO_4_^2−^ ions in solutions are below 50, 50 and 500 mg/L, respectively. The inhibition of scale formation was above 90%. when the concentrations of Mg^2+^, PO_4_^3−^ and SO_4_^2−^ ions in solutions are 60, 60 and 1000 mg/L, respectively. The inhibitory value obtained for MA/APEG-PG-(OH)5 is 88.1%. The results indicate that the interaction of these ions have the ability to affect the inhibition of scale formation to an extent. However, the inhibition of scale formation was still above 77% when the concentrations of Mg^2+^, PO_4_^3−^ and SO_4_^2−^ ions in solutions are 100, 100 and 2000 mg/L, respectively.

**Table 1.** The inhibition of scale formation of MA/APEG-PG-(OH)5 (8 mg/L).

Ions	Concentration (mg/L)							
Ca^2+^	240	240	240	240	240	240	240	240
Mg^2+^	10	20	30	40	50	60	80	100
PO_4_^3−^	10	20	30	40	50	60	80	100
SO_4_^2−^	100	200	300	400	500	1000	1500	2000
CO_3_^2−^	732	732	732	732	732	732	732	732
Inhibition of scale formation (%)	97.2	96.1	94.5	92.4	90.3	88.1	84.6	77.8

### 3.6. Characterization of CaCO_3_ scales

MA/APEG-PG-(OH)n is a structurally well-defined biblock copolymer, one block is PMA.

**Figure 6.**
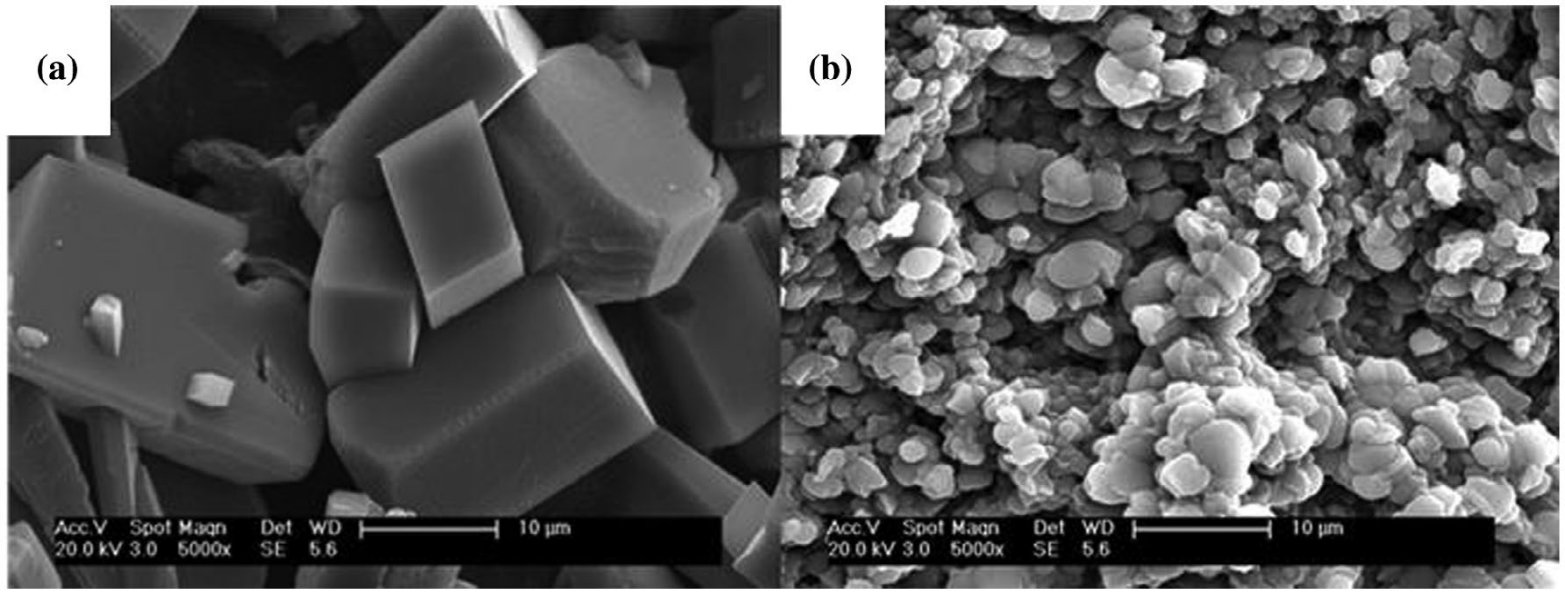
SEM images of the CaCO_3_ crystal formed in the absence of MA/APEG-PG-(OH)5 copolymer (a) and with the presence of 6 mg/L MA/APEG-PG-(OH)5 copolymer (b).

The SEM images for collected CaCO_3_ particle with and without inhibitors are showed in Figure 6. Compared with the two images, both the size and shape of the calcium carbonate precipitation were different due to the addition MA/APEG-PG-(OH)5 copolymer. Without the inhibitor, the CaCO_3_ crystals had regular rhombohedron shape with average particle size of about 10–30 μm (Figure 6(a)). They also had a glossy surface and compact structure. This indicated that the CaCO_3_ crystals without scale inhibitor were mainly composed of calcite, which was the most thermodynamically stable form of CaCO_3_ crystal. In contrast, When the scale inhibitor was added into the sample, the CaCO_3_ crystals loses its sharp edges, and the morphology has been modified from rhombohedron forms to the irregular spherical with relatively loose accumulation. The irregular spherical CaCO_3_ particles diameter are in 1.0–5.0 μm (Figure 6(b)).

The major components of the scale inhibitor were PMA and APEG-PG-(OH)5. During CaCO_3_ crystal growth, the PMA and APEG-PG-(OH)5 group could affect the scale inhibition efficiency by occupying the active sites on the surface of CaCO_3_ crystals and changing the extent of chemical bonding with the surface. In addition, the APEG-PG-(OH)5 group and -COO- group had a good adsorption performance and high chelating ability toward calcium ions to form stable chelation compounds. These would interfere with the nucleation and growth of CaCO_3_ crystals so that the crystals became irregular. The distortion in the CaCO_3_ crystals increased their internal stress, which would lead to crystal fractures and inhibition of deposition of microcrystals. Previous studies suggested that vaterite could be more thermodynamically stable than calcite at certain temperatures or in the presence of some inhibitors [20]. Thus it was illustrated that the vaterite possessed higher thermodynamic stability than calcite in the presence of the MA/APEG-PG-(OH)5 copolymer inhibitor. Because vaterite have a higher solubility product and free energy than calcite, the scale was easy to dissolve and can be washed away by water.

**Figure 7.**
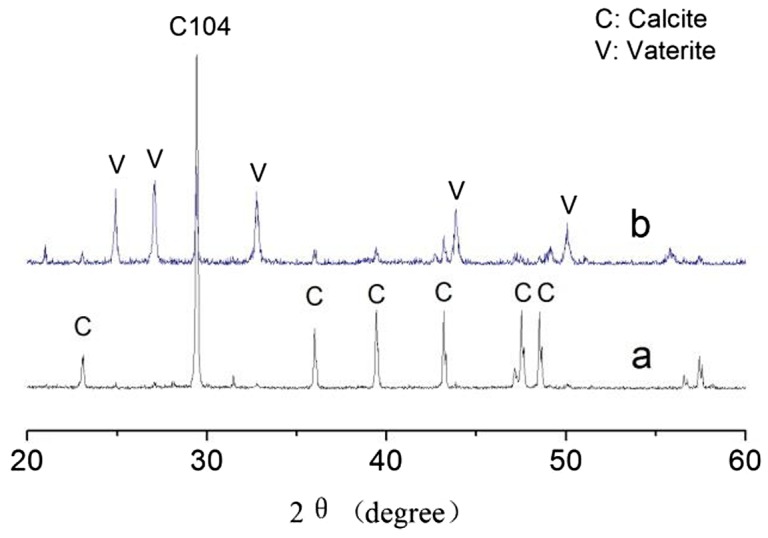
XRD image of the CaCO_3_ crystal formed in the absence of MA/APEG-PG-(OH)5 copolymer (a) and with the presence of 6 mg/L MA/APEG-PG-(OH)5 copolymer (b).

In order to further investigate calcium carbonate crystals, the XRD was measured in Figure 7. In the absence of a copolymer, only sharp calcite reflections appeared, which coincides with the structure of calcite crystal standard substance (Figure 7(a)). This suggested that the morphology of calcium carbonate was mainly a component of calcite. However, when the MA/APEG-PG-(OH)5 copolymer inhibitor was added (Figure 7(b)), both the intense peaks of calcite and vaterite have been discovered by comparison of literature data with their XRD. Therefore, the MA/APEG-PG-(OH)5 copolymer not only can chelate with Ca^2+^, but also modify the formation of CaCO_3_, which illustrated that the studies of XRD also gave consistent results as SEM.

### 3.7. Inhibition mechanism toward CaCO_3_ scale

MA/APEG-PG-(OH)n is a structurally well-defined biblock copolymer, one block is PMA, and the other is polyethylene glycol (PEG) segments. Both PMA and PEG segments are hydrophilic blocks and exist randomly in water. When calcium ions are added into MA/APEG-PG-(OH)n solutions, carboxyl groups in MA/APEG-PG-(OH)n matrixes can recognize and encapsulate or react with positively charged calcium ions either in solutions or on the surface of CaCO_3_ [21,22]. On the other hand, the dendritic pattern polyether has good adsorption effect on calcium ions. As a result, MA/APEG-PG-(OH)n has excellent scale-inhibition performance and exhibit wider application prospect in treatment of industrial circulating cooling water. Chelation and adsorption, between calcium ions and MA/APEG-PG-(OH)n, leads to the spontaneous formation of MA/APEG-PG-(OH)n-Ca complexes. At the same time, water-compatible PEG segments, that is to say, long side chains of MA/APEG-PG-(OH)n, are stable toward aqueous phase because of their high hydrophilic properties [23–25]. As a consequence, the aggregation of CaCO_3_ solid particles is blocked.

## 4. Conclusions

No phosphate and nitrogen free calcium carbonate inhibitor MA/APEG-PG-(OH)5 was successfully synthesized. The structure and thermal property of MA/APEG-PG-(OH)5 were characterized and measured by ^1^H-NMR，GPC and TGA.

MA/APEG-PG-(OH)n copolymer exhibited excellent calcium carbonate inhibition. MA/APEG-PG-(OH)5 displays superior ability to inhibit the precipitation of calcium carbonate, with approximately 97% inhibition at a level of 8 mg/L. Threshold dosage of MA/APEG-PG-(OH)5 is much lower than MA/APEG-PG-(OH)n (*n* = 37,911).

MA/APEG-PG-(OH)5 maintains most of its calcium carbonate inhibition under the conditions of solution pH 7–10, calcium hardness (250–1500 mg/L), HCO_3_^−^concentration (732–1500 mg/L), Temperature (60–90 °C) and Time (10–100 h) and at levels of 0–10 mg/L iron ions in aqueous solutions. The inhibition mechanism of MA/APEG-PG-(OH)n toward CaCO_3_ deposits proposed the formation of the excellent solubility of MA/APEG-PG-(OH)n-Ca complexes due to high hydrophilic PEG segments in the MA/APEG-PG-(OH)n matrix.

SEM and XRD analysis showed that the copolymer of MA/APEG-PG-(OH)5 had a great impact on the morphology and size of the calcium carbonate crystal.

## Disclosure statement

No potential conflict of interest was reported by the authors.

## Funding

The National Natural Science Foundation of China [51077013]; China Postdoctoral Science Foundation [2014M560381]. The Municipal Key Subjects of Environmental Science and Engineering, Nanjing Xiaozhuang University, Nanjing. University Student Technology Innovation Project of Jiangsu Province [201611460008Z]. University Student Technology Innovation Project of Jiangsu Province (School-enterprise cooperation) [201611460085H].
